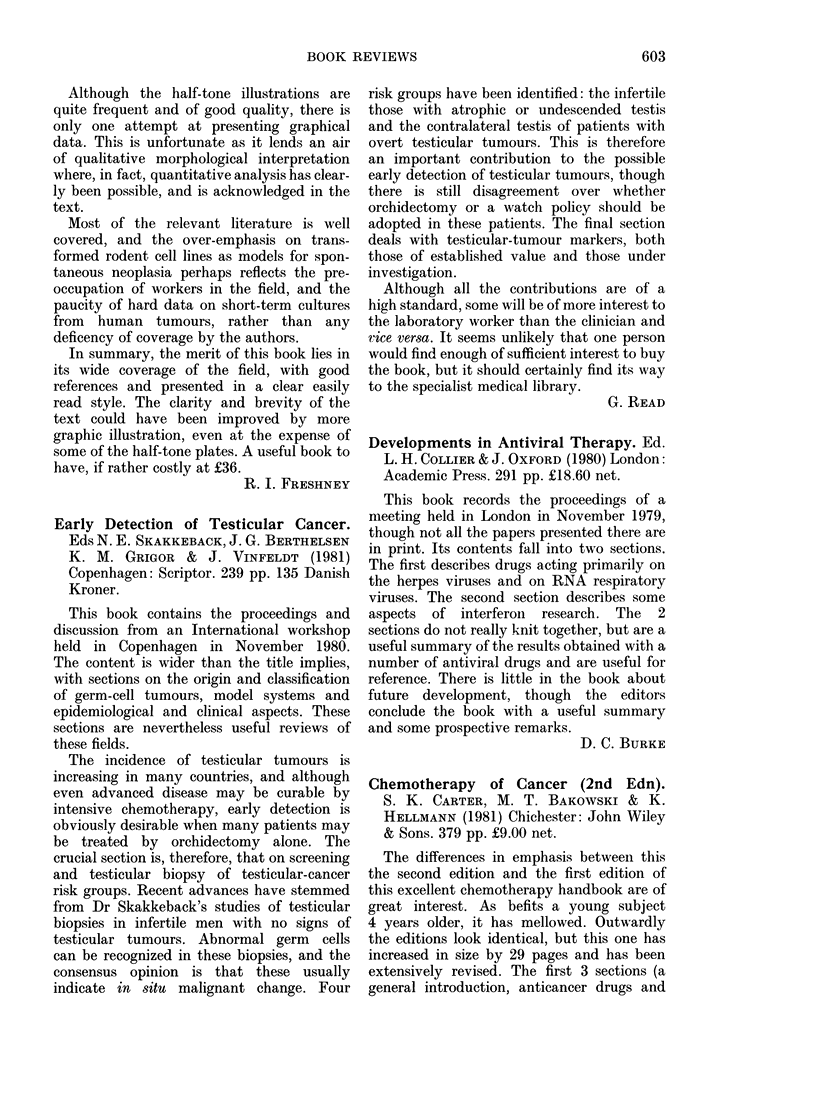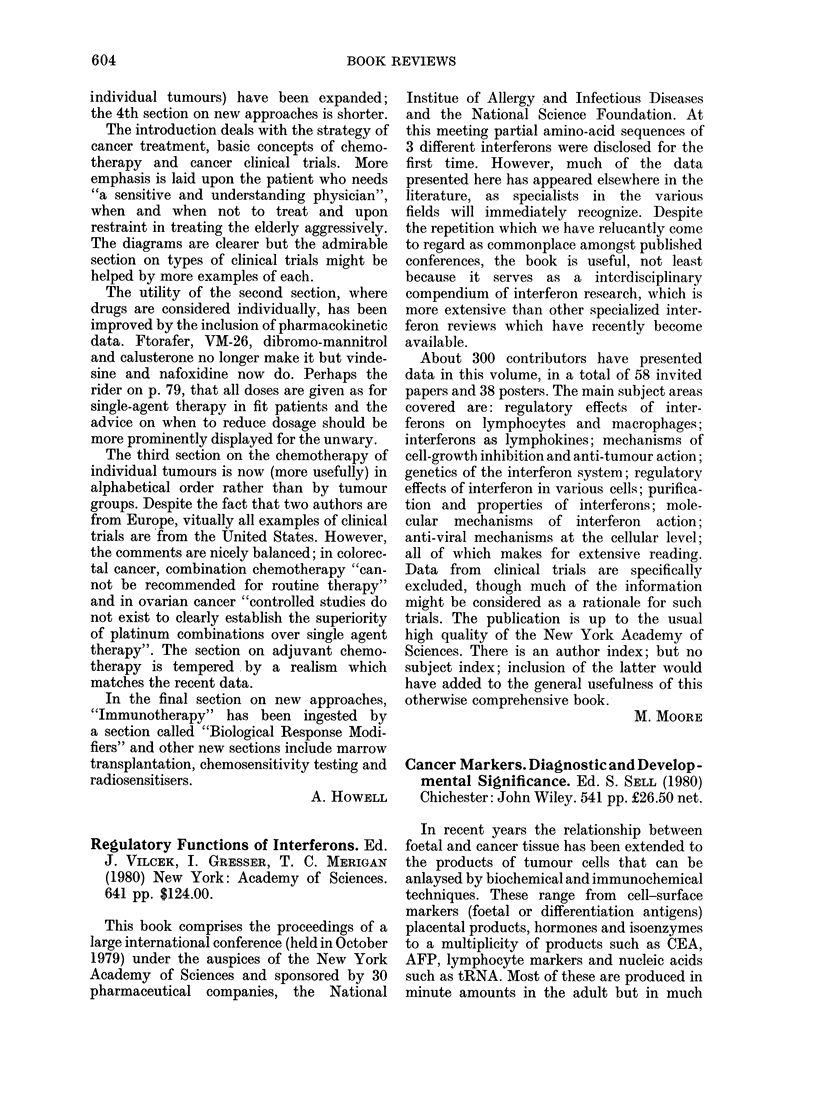# Chemotherapy of Cancer (2nd Edn)

**Published:** 1981-10

**Authors:** A. Howell


**Chemotherapy of Cancer (2nd Edn).** S. K. CARTER, M. T. BAKOWSKI & K. HELLMANN (1981) Chichester: John Wiley & Sons. 379 pp. £9.00 net.

The differences in emphasis between this the second edition and the first edition of this excellent chemotherapy handbook are of great interest. As befits a young subject 4 years older, it has mellowed. Outwardly the editions look identical, but this one has increased in size by 29 pages and has been extensively revised. The first 3 sections (a general introduction, anticancer drugs and individual tumours) have been expanded; the 4th section on new approaches is shorter.

The introduction deals with the strategy of cancer treatment, basic concepts of chemotherapy and cancer clinical trials. More emphasis is laid upon the patient who needs "a sensitive and understanding physician", when and when not to treat and upon restraint in treating the elderly aggressively. The diagrams are clearer but the admirable section on types of clinical trials might be helped by more examples of each.

The utility of the second section, where drugs are considered individually, has been improved by the inclusion of pharmacokinetic data. Ftorafer, VM-26, dibromo-mannitrol and calusterone no longer make it but vindesine and nafoxidine now do. Perhaps the rider on p. 79, that all doses are given as for single-agent therapy in fit patients and the advice on when to reduce dosage should be more prominently displayed for the unwary.

The third section on the chemotherapy of individual tumours is now (more usefully) in alphabetical order rather than by tumour groups. Despite the fact that two authors are from Europe, vitually all examples of clinical trials are from the United States. However, the comments are nicely balanced; in colorectal cancer, combination chemotherapy "cannot be recommended for routine therapy" and in ovarian cancer "controlled studies do not exist to clearly establish the superiority of platinum combinations over single agent therapy". The section on adjuvant chemotherapy is tempered by a realism which matches the recent data.

In the final section on new approaches, "Immunotherapy" has been ingested by a section called "Biological Response Modifiers" and other new sections include marrow transplantation, chemosensitivity testing and radiosensitisers.

A. Howell